# Supplementary material for: Attention strengthens across-trial pre-stimulus phase coherence in visual cortex, enhancing stimulus processing
Source: Sci Rep. 2020 Mar 16;10:4837. doi: 10.1038/s41598-020-61359-7 (PMC7076023; doi:10.1038/s41598-020-61359-7)
Supplement: Supplementary file 1 — Supplementary figures. [file 41598_2020_61359_MOESM1_ESM.docx]

# Supplementary figures

Attention strengthens across-trial pre-stimulus phase coherence in visual cortex, enhancing stimulus processing

Behzad Zareian, Kourosh Maboudi, Mohammad Reza Daliri, Hamid Abrishami Moghaddam, Stefan Treue, Moein Esghaei


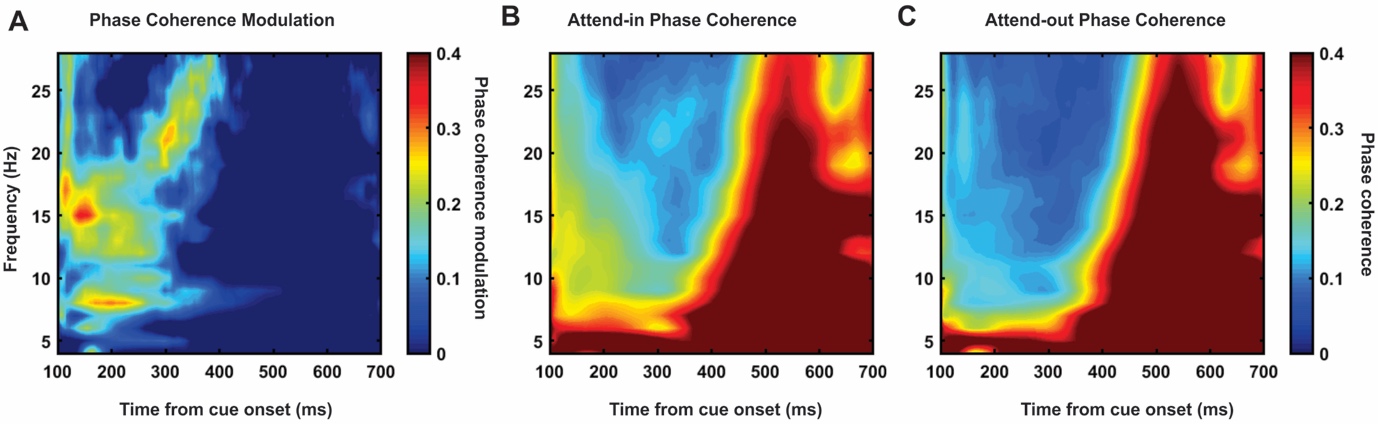


**Figure S1. Full frequency range for phase coherence modulation (PCM).** **Related to Figure 1.** (A) PCM for frequencies between 2 to 30 Hz. (B) Same as A, but for the average phase coherence across sites only for attend-in trials. (C) Same as B, but for the average phase coherence across sites only for attend-out trials.

**
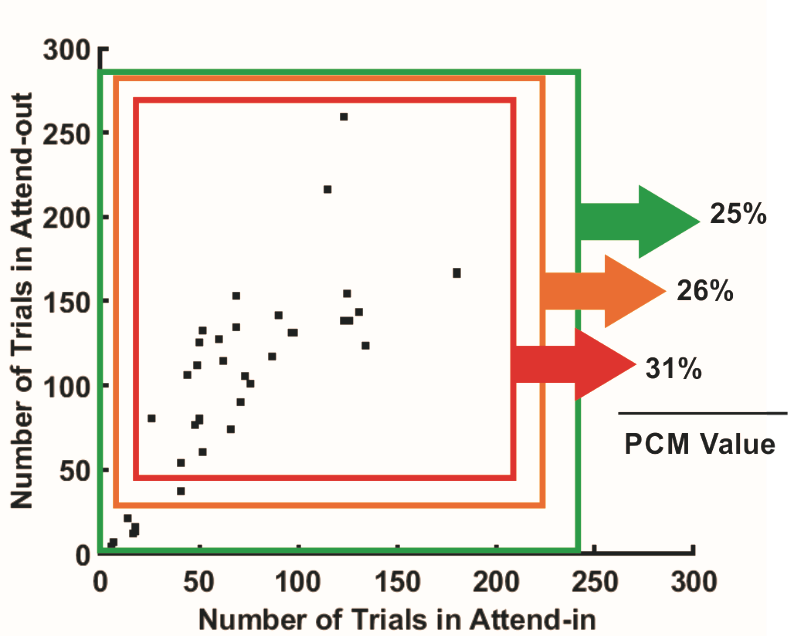
**

**Figure S2.** **Phase coherence modulation (PCM) at (200 ms, 8 Hz) including (excluding) the sites with low number of trials in both attention conditions. Related to Figure 1.** Each point in the scatter plot corresponds to one site (from a total of 41 sites). The horizontal axis shows the number of trials in the attend-in condition and the vertical axis shows the attend-out condition. These results show that the PCM effect is observed independent of the selection of sites. The red square shows the set of sites focused for the analyses carried out here (31 sites).


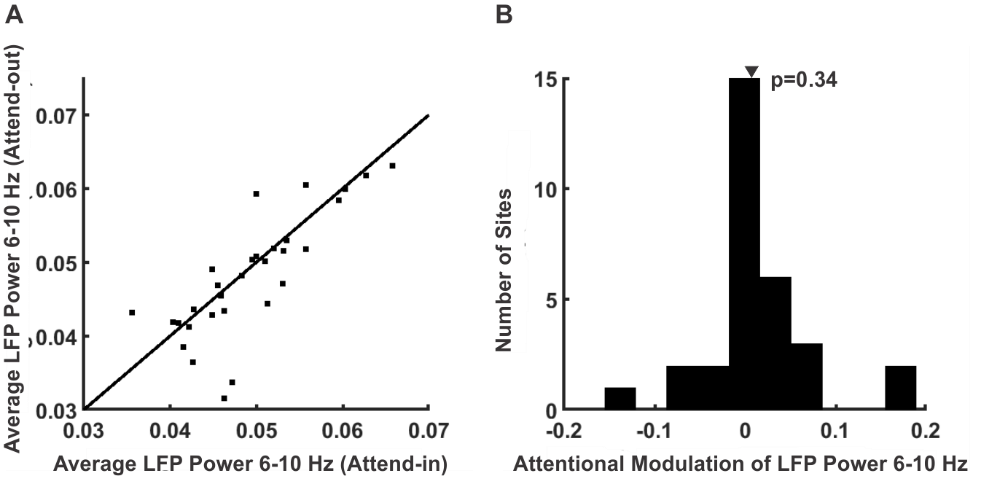


**Figure S3.** **LFP power analysis within the pre-stimulus interval**. **Related to Figure 1.** (A) The scatter plot shows the averaged LFP power across trials for 6-10 Hz band during 100-700 ms after cue onset. Each point corresponds to one site. The horizontal axis shows the average LFP power across trials for the attend-in subset of trials in each session and the vertical axis shows corresponds to the attend-out condition. (B) The histogram shows the attentional modulation of power in the same period showing no bias to positive values (p-value=0.34; ttest).


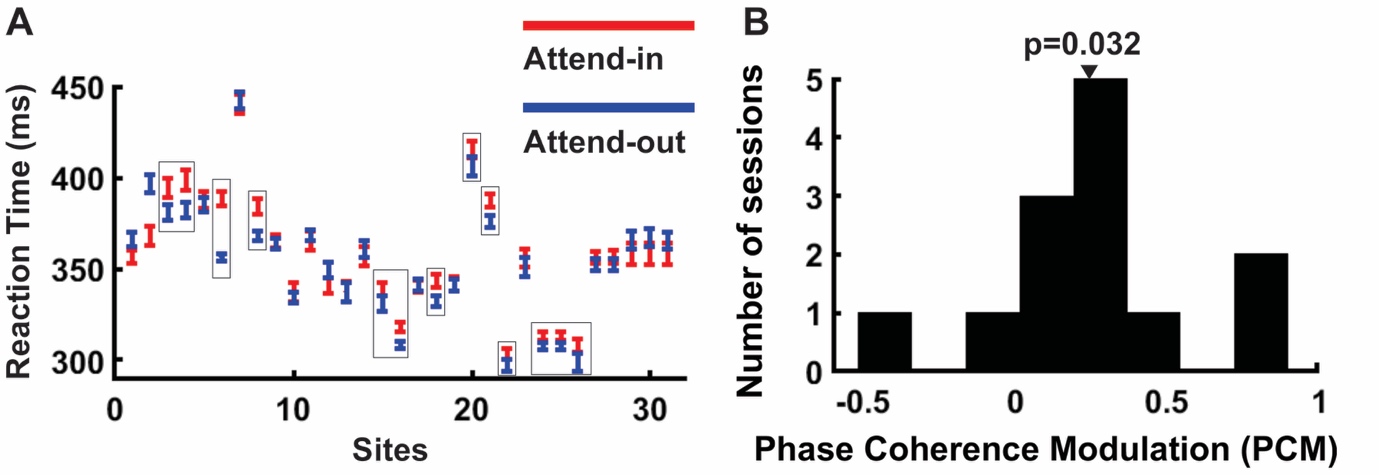


**Figure S4.** **Control analysis addressing the difference of arousal level. Related to Figure 1.** (A) X-axis indicates different sites and Y-axis represents the reaction time. Monkey responds faster in attend-out condition in sites indicated by rectangle. (B) PCM for sites indicated by rectangles in (A) (p-value<0.001; ttest). Bars show the standard error of the mean.


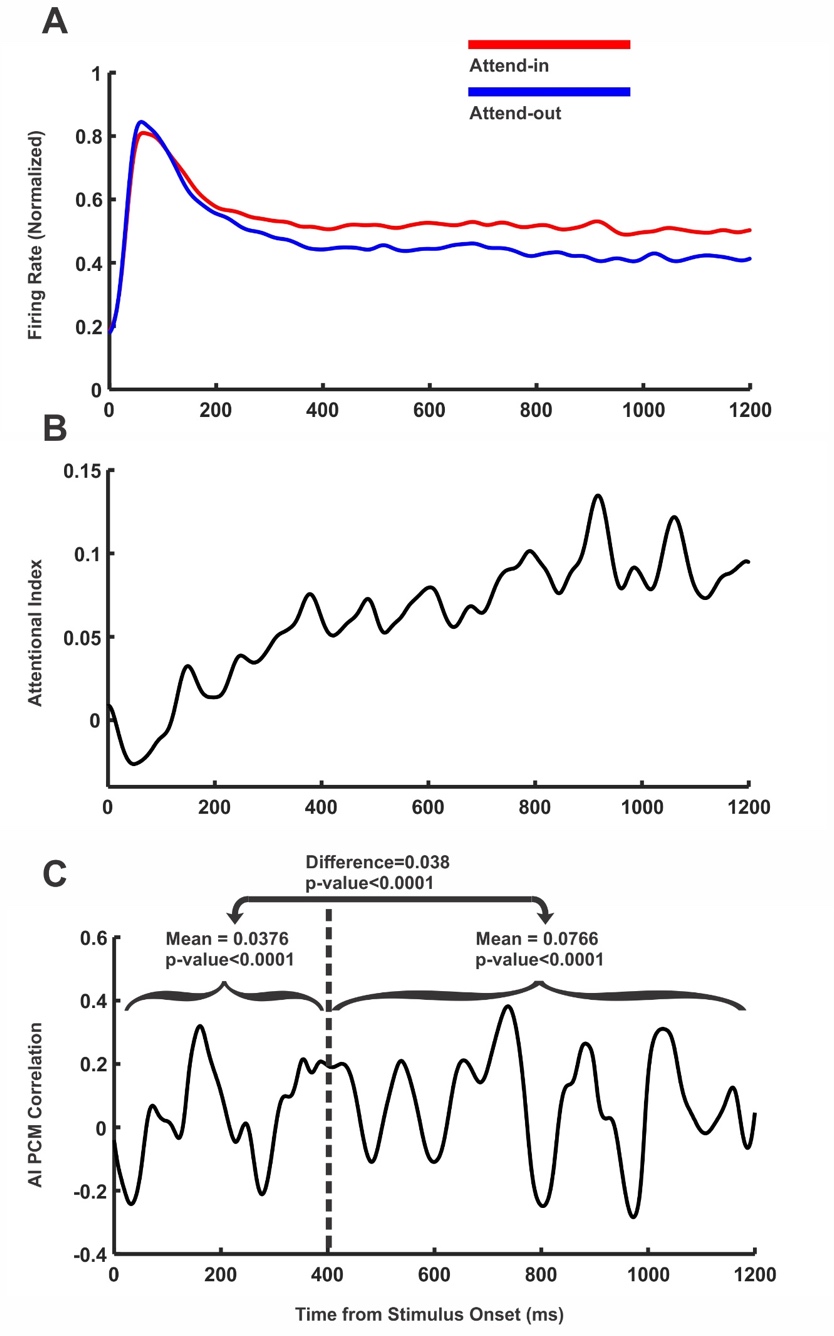


**Figure S5. Post-stimulus traces of spike rate, AI and AI-PCM correlation. Related to Figure 3.** (A) Changes in firing rate after stimulus onset in attend-in (red) and attend out (blue) trials. (B) Time-resolved changes in the attentional index ( (attend-in – attend out)/(attend-in + attend out) ) following the stimulus onset. (C) correlation between pre-stimulus PCM (at 200 ms, 8 Hz) and post-stimulus attentional index. Both the immediate post-stimulus interval (1-400 ms post-stimulus) and later times (401-1200 ms) show a significant positive skewness (p-value<0.0001; ttest), while the late interval shows a significantly larger correlation compared to the early interval (p-value<0.0001; two sample ttest).
